# General value functions for fault detection in multivariate time series data

**DOI:** 10.3389/frobt.2024.1214043

**Published:** 2024-03-13

**Authors:** Andy Wong, Mehran Taghian Jazi, Tomoharu Takeuchi, Johannes Günther, Osmar Zaïane

**Affiliations:** ^1^ Computing Science Department, Alberta Machine Intelligence Institute, University of Alberta, Edmonton, AB, Canada; ^2^ Information Technology R&D Center, Mitsubishi Electric Co., Kamakura, Japan

**Keywords:** reinforcement learning, general value functions, outlier detection, fault detection, temporal difference (TD) learning

## Abstract

One of the greatest challenges to the automated production of goods is equipment malfunction. Ideally, machines should be able to automatically predict and detect operational faults in order to minimize downtime and plan for timely maintenance. While traditional condition-based maintenance (CBM) involves costly sensor additions and engineering, machine learning approaches offer the potential to learn from already existing sensors. Implementations of data-driven CBM typically use supervised and semi-supervised learning to classify faults. In addition to a large collection of operation data, records of faulty operation are also necessary, which are often costly to obtain. Instead of classifying faults, we use an approach to detect abnormal behaviour within the machine’s operation. This approach is analogous to semi-supervised anomaly detection in machine learning (ML), with important distinctions in experimental design and evaluation specific to the problem of industrial fault detection. We present a novel method of machine fault detection using temporal-difference learning and General Value Functions (GVFs). Using GVFs, we form a predictive model of sensor data to detect faulty behaviour. As sensor data from machines is not i.i.d. but closer to Markovian sampling, temporal-difference learning methods should be well suited for this data. We compare our GVF outlier detection (GVFOD) algorithm to a broad selection of multivariate and temporal outlier detection methods, using datasets collected from a tabletop robot emulating the movement of an industrial actuator. We find that not only does GVFOD achieve the same recall score as other multivariate OD algorithms, it attains significantly higher precision. Furthermore, GVFOD has intuitive hyperparameters which can be selected based upon expert knowledge of the application. Together, these findings allow for a more reliable detection of abnormal machine behaviour to allow ideal timing of maintenance; saving resources, time and cost.

## 1 Introduction

Automation and robotics allow manufacturers to produce goods far more cheaply and consistently than hand-made products. With the advances in automated industrial production processes, the cost of downtime has grown proportionally. While machines can be designed for constant up-time, they will still degrade over time. As degradation is dependent on many variables, it is nearly impossible to accurately predict when the machine will break without actively monitoring its status. It is therefore imperative to shut machines down in a controlled way in order to maintain them before they break uncontrolled.

Deciding when to shut down machines for maintenance is still a field of ongoing research (Si et al., 2011; Lei et al., 2018). Performing maintenance only when the machine is in actual need is called condition-based maintenance (CBM) (Jardine et al., 2006). CBM therefore prevents unnecessary downtime and the replacement of parts that still have a long lifespan, ultimately saving on maintenance costs. This maintenance approach includes the tasks of fault detection and fault classification (called *diagnostic* problems), along with predicting the remaining useful life and the prediction of probability of failure (called *prognostic* problems). With these four values, it is possible to make informed decisions about when maintenance is necessary. In this paper, we focus on the task of fault detection.

The advantages of CBM come at the cost of needing data to access the state of the machine—the process of collecting data about the machine while it runs is called Condition Monitoring (CM). As the data are constantly collected while the machine is running, it is time series data. Examples of CM data include, but are not limited to: position, speed, force, vibration, and temperature. The need for data is an Achilles heel for CBM; to collect data, appropriate sensors are necessary. The performance of CBM is highly correlated with the availability and quality of data.

One way to ensure sufficient data for CBM is the installation of sensors. Sensors can be targeted towards a certain failure mode; e.g., a thermocouple can be installed and monitored for a process that is prone to overheat. While the installation of sensors ensures high quality data that caters to the exact need of CBM, it is also a costly solution. A solution that is more cost efficient is the use of existing sensors to gather data. Most industrial processes are already equipped with sensors that are necessary for their functioning; e.g., sensors for feedback control, machine calibration, or sensors that are installed to access the product quality. If these sensors can be used to infer machine degradation, they provide a cost efficient solution to CBM.

The use of existing sensors for anomaly detection is a field of active research and a review of supervised, semi-supervised, and unsupervised anomaly detection can be found in Hodge and Austin (2004) and Pimentel et al. (2014). Furthermore, Gupta et al. (2014) provided a review for (classical) outlier detection in time series data. More recently, Riazi et al. (2019) published a comprehensive study on data-driven outlier detection that compared different classical machine learning algorithms.

**FIGURE 1:**
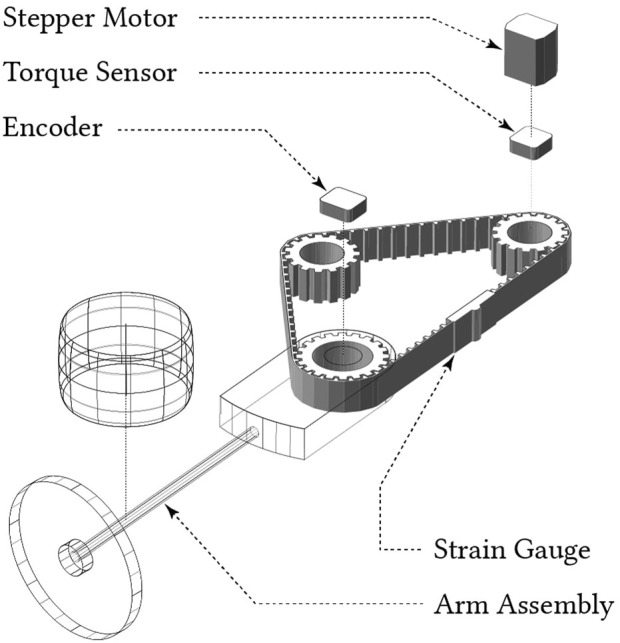
A diagram showing the construction of the robot arm testbench, from which data arising from normal and faulty operation was collected.

Riazi et al. (2019) also collected operating data from a lab-scale robotic arm, depicted in Figure 1. This data was labeled, indicating whether the arm was operating normally or if there was some fault in its operation. Within that study, machine fault detection was framed as a semi-supervised outlier detection problem. In the training phase, a multivariate semi-supervised outlier detection algorithm learns a *boundary of normality* from a random sample of normal operating data. In the testing phase, the algorithm evaluates whether new, unseen samples lie within or beyond the boundary, and classifies it as an inlier or outlier accordingly.

Previous work on using machine learning for outlier detection were predominantly based on supervised and unsupervised machine learning algorithms (Riazi et al., 2019). While these approaches demonstrated good performance, they fail to take the temporal structure of the data into account. Many approaches, especially those based on deep learning, assume the data to be i.i.d., which is not the case in this application. New data are highly correlated to previous data, resulting in a data distribution that can be seen as the result of Markovian sampling (Arnold, 1970). For this reason, we suggest the use of temporal-difference (Sutton, 1988) based methods, as predictions learned with these methods have already shown their potential in industrial processes (Günther et al., 2016) and they are a well-fitted approach to this domain.

Our paper extends the work of Riazi et al. (2019) in two major ways. First, we modify the random sampling and validation techniques from machine-learning practice to better represent real-world deployments of fault detection. Therefore, we ensure that *normal* training data occurs strictly prior to *normal* testing data. Second, we implement three state-space models for time-series data, including a new algorithm based on temporal-difference learning, named General Value Function Outlier Detection (GVFOD). GVFOD is a novel method of applying general value functions for fault detection. We investigate whether these strictly temporal methods can outperform general multivariate methods. To the best of our knowledge, this is the first example of using predictions which are learned by the methods of temporal-difference learning to detect faulty behaviour in data streams from machine data. It is also one of few studies that show how reinforcement learning methods can be leveraged in industrial processes.

Leveraging machine learning to use data from existing sensors promises to address one of the most important challenges for CBM. By applying reinforcement learning techniques to take the temporal structure of machine data into account, we extend the current state of the art and demonstrate how this approach successfully detects outliers in data. We therefore provide a cost-effective solution to fault detection in order to enable automated CBM in real-world applications.

## 2 Background and methods

This study considers outlier detection as a semi-supervised problem. In a real-world situation, these algorithms would be employed in situations where known-good (or known-mostly-good) operational data is available, and where it is tolerable for fault detection to be enabled only after a period of known-good operation. It is also permissive of applications where faulty data is not available (or mixed within the normal data).

In semi-supervised outlier detection, the training dataset consists only of *normal* records, while the testing dataset has a mix of *normal* and *abnormal* records. An algorithm will first learn a model of normality using the training data. The algorithm will then assign a numerical outlier score to each sample in the training dataset. Based on the empirical distribution of training outlier scores, a threshold of normality can be determined. When the model is used to calculate outlier scores on the testing data, the algorithm classifies each sample as either an inlier or an outlier. We use the convention where scores larger than the threshold are outliers, and scores smaller than the threshold are inliers.

The outlier threshold is dependent on a common hyperparameter across all algorithms, the *contamination ratio*, CR ∈ (0, 0.5]. The contamination ratio defines the probability that a future inlier will be incorrectly classified as an outlier; and equivalently, the proportion of training data that lies beyond the outlier threshold. In this study, all algorithms had contamination ratios of 5%; and thus, predicted outliers are those with outlier scores (*OS*) above the 95th percentile.

### 2.1 Multivariate outlier detection

We evaluate the best multivariate outlier detection techniques from Riazi et al. (2019) and Gupta et al. (2014). The methods in both papers have been applied successfully to similar data sets, validating this decision. They span multiple classes of anomaly detectors, including probabilistic, distance/density, reconstruction, and domain-based methods. The algorithms include.

• Isolation forest (IForest) (Liu et al., 2008)
• One-class support vector machine (OCSVM) (Schölkopf et al., 1999)
• Local outlier factor (LOF) (Breunig et al., 2000)
• Angle-based outlier detection (ABOD) (Kriegel et al., 2008)
• k^th^-nearest neighbour (KNN) (Ramaswamy et al., 2000)
• Histogram-based outlier score (HBOS) (Goldstein and Dengel, 2012)
• Minimum covariance discriminant (MCD) (Rousseeuw, 1984)
• Principal components analysis (PCA) (Shyu et al., 2003) and all are implemented in the PyOD (Zhao et al., 2019) package.

### 2.2 State-space models for temporal outlier detection

Furthermore, we include temporal algorithms to make our comparisons more meaningful. Two existing state-space models are used for outlier detection:

• Markov chain (MarkovChain) (Ye et al., 2000)
• Hidden Markov model (HMM) (Smyth, 1994; Schreiber, 2017)

**FIGURE 2:**
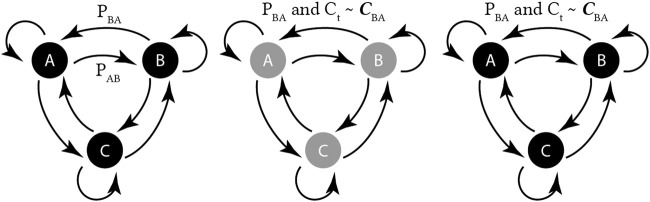
Overview of state-space temporal outlier detection approaches. (left) a Markov chain with three observable states. (middle) A hidden Markov model, with three hidden (greyed-out) states, and observable cumulants. (right) An MDP with three observable states, and a cumulant is provided with each state-transition. Actions omitted for clarity.

Examples of these are shown in Figure 2.

In a Markov chain (Figure 2, left), the world is described sequentially, where at each time step *t*, transitions are made from state to state, with $x_{t},x_{t+1}\in X$ . These transitions are completed stochastically, following some transition probability $P:X\times X\mapsto R^{+}$ , which is trivial to estimate from data.

In a hidden Markov model (HMM) (Figure 2, middle), the same transitions are made, except the underlying states ***x***_*t*_ cannot be observed. Instead, a finite number of the hidden states are inferred from the sensor observations. The Baum-Welch algorithm is used to estimate the states, state-transition probabilities *P*, and emission probabilities *C*. In general, HMM estimation is a computationally slow process (Gupta et al., 2014).

In a Markov decision process (MDP) (Figure 2, right), an agent takes an action $A_{t}\in A$ , which causes a similar transition from the observed ***x***_*t*_ to ***x***_*t*+1_. The transition probabilities in an MDP are action-dependent, with $P^{\prime }:X\times A\times X\mapsto R^{+}$ . The agent also receives a reward as feedback for its choice of action. The agent’s choice of action, *A*_*t*_, is determined by a probability function $\pi :X\times A\mapsto R^{+}$ , known as a *policy*.

### 2.3 Prediction learning methods and general value functions

This paper uses a prediction learning method called General Value Functions (GVF) (White, 2015). GVFs learn to make predictions solely by interacting with the environment—they are based on observations.

We learn predictions by forming predictive questions (Sutton et al., 2011) about our sensor signals, which we phrase as GVFs—predictions about a signal of interest, or *cumulant*, *C*, from the environment over some time-scale or horizon defined by *γ* ∈ [0, 1), and a behaviour policy *π*. The discounted future sum of the cumulant is known as the *return*, $G_{t}=\sum_{k=0}^{\infty }\gamma^{k}C_{t+k+1}$ . A GVF is the expected return of this cumulant: $V\left(x;\pi ,\gamma ,C\right)=E\left[G_{t}|X_{t}=x\right]$ , which can be estimated using incremental online learning methods, such as TD learning (Sutton and Barto, 2018). The learning algorithm, TD(*λ*), and the TD-error, *δ*, are shown in Algorithm 1, lines 3–8. TD(*λ*) uses a step-size *α* and a trace-decay parameter *λ*. The step-size, along with the TD-error, determine the magnitude of GVF updates. The trace-decay parameter, *λ*, determines how much the current TD-error will update GVF estimates in previously visited states.

Like in Markov chains, GVFs use sensory observations as state. Features are constructed from observations using tile coding. Tile coding $\phi :R^{k}\mapsto {\left\{0,1\right\}}^{d}$ , maps observations from a bounded continuous space into a sparse binary feature vector, and is a common technique from Sutton and Barto (2018) Tile coding has two hyperparameters. divs_per_dim $\in N^{k}$ defines how many divisions are made in each dimension—resulting in a grid of tiles. The size of a tile limits the extent to which learning on one state can generalize to neighboring states. n_tilings ∈N determines the number of offset tilings to use. It also determines the number of ones in the feature vector. Combined, divs_per_dim and n_tilings determine the specificity of the features.

In this paper, we leverage a signal, called Unexpected Demon Error (UDE) (White, 2015). This signal is a measure for unexpected changes in the TD-error due to changes in the environment. The UDE can be thought of as *surprise* (Günther et al., 2018) and is potentially a useful measure for outlier detection. Mathematically, the UDE for a sensor is calculated as

$$UDE\left(t,\delta \right)=\left|\frac{\frac{1}{\beta }\sum_{i=t-\beta }^{t-1}\delta_{i}}{\sigma_{\delta ,\left[t\right]}+\epsilon }\right|$$ (1)

where *β* is a sliding window width that defines the timescale of recent TD-errors; *σ*_***δ***,[*t*]_ is the standard deviation of TD-errors prior to *t*; and *ϵ* is a small constant to prevent division by zero. Intuitively, the UDE learns the recent distribution of TD-errors and will therefore only spike if the environment provides the learning agent with signals that are new.

We use linear function approximation in conjunction with tile coding to approximate the true value function. The form of the GVF estimate is $\hat{v}\left(x_{t}\right)=w^{⊺}\cdot \phi \left(x_{t}\right)$ , where ***w*** are the weights to be learned, ***x*** are the sensor values, and ***ϕ*** is the tile coder which creates the linear basis functions of $\hat{v}$ . When combined with linear function approximation, temporal-difference learning scales linearly with the number of features, making it an excellent candidate for real-world tasks (Sutton and Barto, 2018).

## 3 General value function outlier detection (GVFOD)

Given a training dataset $\left\{X_{i}|X_{i}\in R^{T\times k};i\in \left[n\right]\right\}$ , GVFOD requires that the time series are ordered chronologically, so they can be unrolled into a single time series $X\in R^{\left(nT\right)\times k}$ . For each of the *k* sensors, a GVF is learned, which combined serve as a model of normality. The learning phase then proceeds as in Algorithm 1. Evaluation of test data can be seen in Algorithm 2.

Algorithm 1 General Value Function Outlier Detection (Training).

1: Using training data $X\in R^{\left(nT\right)\times k}$

2: Initialize weights ***w*** = 0^*d*×*k*^, traces ***z*** = 0^*d*×*k*^, TD-errors ***δ*** = 0^(*nT*)×*k*^, scalar step size *α*, surprise $UDE\in R^{\left(nT\right)\times k}$ , outlier scores $OS\in R^{n}$ , and scalar outlier threshold OS*
3: Repeat for each sensor *j* ∈ [*k*]:
4:  Observe ***x***_*t*_ = ***x***_0_ := ***X***_0,:_
5:  For each subsequent observation ***x***_*t*+1_ in ***X***
6: $\delta \leftarrow x_{t+1,j}+\gamma w_{j}^{⊤}\cdot \phi \left(x_{t+1}\right)-w_{j}^{⊤}\cdot \phi \left(x_{t}\right)$

7:   ***z***_***j***_ ← *γλ* ***z***_***j***_ + ***ϕ*** (***x***_*t*_)
8:   ***w***_***j***_ ← ***w***_***j***_ + *αδ* ***z***_***j***_
9: Repeat for each sensor *j* ∈ [*k*]:
10:  Observe ***x***_*t*_ = ***x***_0_ := ***X***_0,:_
11:  For each subsequent observation ***x***_*t*+1_ in ***X***
12: $\delta_{tj}\leftarrow x_{t+1,j}+\gamma w_{j}^{⊤}\cdot \phi \left(x_{t+1}\right)-w_{j}^{⊤}\cdot \phi \left(x_{t}\right)$

13:   *UDE*_*tj*_ ← *UDE*(*t*, ***δ***_[*t*],*j*_) # See Eq. 1
14: Repeat for each period *i* ∈ [*n*]:
15: *OS*_*i*_ ← *OS*_*GVFOD*_(*i*, ***UDE***) # See Eq. 2
16: OS* ← *quantile*(***OS***, (1 − *α*))
17: **return** ***w***, OS*

Algorithm 2 General Value Function Outlier Detection (Inference).

1: Using weights ***w***, an outlier threshold OS*, and testing data $X\in R^{\left(nT\right)\times k}$

2: Initialize TD-errors ***δ*** ∈ 0^(*nT*)×*k*^, surprise $UDE\in R^{\left(nT\right)\times k}$ , outlier scores $OS\in R^{n}$ , and outlier classifications *y* ∈ {0,1}^*n*^
3: Repeat for each sensor *j* ∈ [*k*]:
4:  Observe ***x***_*t*_ = ***x***_0_ := ***X***_0,:_
5:  For each subsequent observation ***x***_*t*+1_ in ***X***
6: $\delta_{tj}\leftarrow x_{t+1,j}+\gamma w_{j}^{⊤}\cdot \phi \left(x_{t+1}\right)-w_{j}^{⊤}\cdot \phi \left(x_{t}\right)$

7:   *UDE*_*tj*_ ← *UDE*(*t*, ***δ***_[*t*],*j*_) # See Eq. 1
8: Repeat for each period *i* ∈ [*n*]:
9:  *OS*_*i*_ ← *OS*_*GVFOD*_(*i*, ***UDE***) # See Eq. 2
10:  *y*_*i*_ ← [*OS*_*i*_ > OS*]
11: **return** ***OS***, ***y***_***i***_

Compared to a typical application of TD(*λ*) and UDE, GVFOD is now an offline learning algorithm, requiring two passes through the training data. The first pass (lines 3–8) learn the weights in order to make accurate predictions about the data. In the second pass through the data (lines 9–13), the weights are fixed, and no model updates occur. The computational complexity, like TD(*λ*), scales linearly with the number of features (*d*), the experience (*nT*) and the number of sensors (*k*).

The outlier scores for a single period are calculated as the average surprise (UDE) over the *k* sensors and *T* time steps. For a multivariate time series ***X***_*i*_, the scalar outlier score is

$$OS_{\mathrm{GV FOD}}\left(X_{i}\right)=\frac{1}{Tk}\overset{\left(i+1\right)T-1}{\underset{t=iT}{\sum }}\underset{j\in \left[k\right]}{\sum }UDE_{j}\left(t\right)$$ (2)

when the model encounters surprising data, the outlier score will increase, indicating anomalous behaviour. Because weights are fixed during inference—no learning is occurring, and the *UDE* and corresponding *OS*_*GVFOD*_ remain elevated during the entire duration of abnormal behavior. This contrasts with the original online implemention of *UDE* (Günther et al., 2018), where *UDE* eventually dies down as the learners adapt to new operating conditions.

There are many hyperparameters for GVFOD. divs_per_dim and n_tilings were previously discussed for tile coding. The discount rate *γ* ∈ [0, 1), step-size *α* ∈ (0, *n*_*tilings* ^−1^], and trace-decay parameter, *λ* ∈ [0, 1), are part of the original *TD*(*λ*) algorithm. β∈N determines the sliding window width for UDE calculation. Notice also that *λ* and *α* are used only in the training phase, and all other hyperparameters are used in both training and testing. Albeit numerous, selection of these hyperparameters is straightforward and demonstrated in Section 5.

A Python implementation of GVFOD is provided on GitHub.

## 4 Experimental setup

A full description of the robotic arm testbench in Figure 1 can be found in Lipsett et al. (2019). The robot arm is mounted on a stable platform, with the arm assembly rolling horizontally on a steel plate. The assembly moves between two angular positions with a period of 10*s*. There are a total of three sensors: for arm position, motor torque, and belt tension; sampling data at 200*Hz*.

**FIGURE 3:**
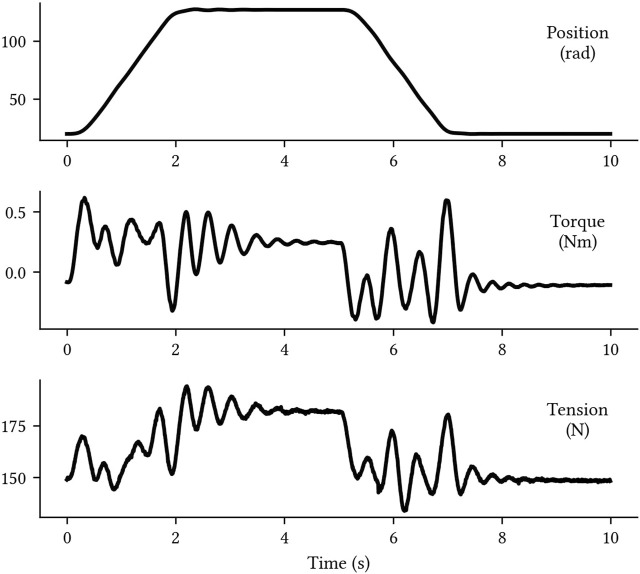
A sample of *normal* robot arm data.

A summary of the available data are presented in Table 1, and a sample of *normal* data can be seen in Figure 3. Each sample, $X_{i}\in R^{\mathrm{2000\times 3}}$ , is a multivariate time series containing the observations from each sensor for the entire period. Three sensors are installed - an encoder for arm position, a strain gauge on the motor output shaft to measure torque, and a strain gauge on the rubber belt to measure tension. To collect *normal* operating data, the arm was allowed to operate undisturbed for roughly 22 h, creating a total of 7193 samples. The machine was then stopped, and modified, to create a faulty operating condition. Five of these *abnormal* datasets were collected: three contain data from varying the belt tension level; one contains data from a sandy rolling surface; and one contains data from both high belt tension and high operating temperature^1^. The data was cleaned by removing the startup and shutdown data, including the first and last periods if they were incomplete. In all subsequent experiments, we use *Loose L1* data as the sole source of abnormal data, since its operation is most similar to *normal* data, and demonstrates the worst-case performance on this collection of data (Riazi et al., 2019).

**TABLE 1:** Robot arm datasets.

Case	Samples	Belt tension (N)	Description
**Normal**	**7193**	**160**	Normal operation.
Loose L2	180	120	Varying belt tension levels.
**Loose L1**	**182**	**140**	
Tight	194	180	
Sandy	183	160	Sand on rolling surface.
HighT	210	180	Tight belt *and* heated with two incandescent floodlights.

Bolded values indicate the subset of data that was used in this experiment.

Furthermore, we have split each *normal* and *abnormal* dataset into two parts. The first part—the *tuning* dataset(s)—contains roughly the first half of available data: 4197 samples of *normal* data, and the first half of each of the *abnormal* classes. The *tuning* dataset is used for hyperparameter selection^2^ using the Tree of Parzen Estimators (TPE) algorithm (Bergstra et al., 2011). The second part—the *evaluation* dataset(s)—consists roughly of the latter half of data: 4196 samples of *normal* data, and the last half of each of the *abnormal* classes. It is used solely for evaluation. In this way, there is minimal overlap in *normal* data between the *tuning* and *evaluation* datasets, and no overlap for the *abnormal* data. We reiterate that this does not follow the typical train-validate-test splitting of typical machine learning problems, due to restriction that training data consists only of *normal* data, and must precede the *normal* data in the test set. We instead choose train-validate-test datasets that minimize the amount of data overlap between folds, while ensuring data is contiguous within folds (Wong, 2021).

The values that were found by TPE for GVFOD are shown in Table 2. The default and TPE search values for all other algorithms are shown in Table 3. The TPE algorithm evaluated 400 candidate parameter combinations and picked the best, using the *tuning* dataset. With the exception of the HMM model, this heuristic search was completed in a similar manner for all other outlier detection algorithms, and all were limited to the same number of trials. Expert selection of the only HMM hyperparameter—the number of hidden states—was used in all experiments due to computational constraints.

**TABLE 2:** GVFOD hyperparameters.

Name	Symbol	Value (search)	Value (expert)
divs_per_dim		(7,7,2)	(10,10,10)
n_tilings	*m*	2	10
discount_rate	*γ*	0.96	0.90
step_size	*α*	0.052	0.001
lambda	*λ*	0.209	0.1
beta	*β*	888	250

**TABLE 3:** Algorithms and hyperparameters used.

Algorithm	Hyperparameter	Value (default)	Value (search)
IForest	n_estimators	100	14
	max_features	1.0	0.88
	bootstrap	False	True
OCSVM	kernel	rbf	sigmoid
	nu	0.5	0.996
	gamma	0.05	4.280e-05
	coef0	N/A	0.103
LOF	n_neighbors	20	500
	metric	Euclidean	Chebyshev
ABOD	n_neighbors	5	92
kNN	n_neighbors	5	500
	metric	Euclidean	Chebyshev
HBOS	n_bins	10	10
	alpha	0.1	0.827
	tol	0.5	0.754
MCD	support_fraction	0.5	0.714
PCA	n_components	All	3
	weighted	True	False
	whiten	False	True
Markov Chain	divisions	N/A	8
HMM	n_states	N/A	8
GVFOD	divs_per_dim	N/A	[7, 7, 2]
	numtilings		2
	discount_rate		0.96
	step_size		0.052
	lambda		0.209
	beta		888

In order to realistically evaluate the performance of the multivariate and temporal outlier detection methods for machine fault detection, we design our experimental training and testing data to emulate a real CBM implementation. Samples in a single dataset are always ordered consecutively. Data in the training set consists only of *normal* samples, collected immediately prior to *normal* samples in the testing set. The size of the training set is variable to see how algorithms react to varying training data availability. The testing set contains both *normal* samples and ones from a single fault class. In the testing set, the *abnormal* data are appended to the end of the *normal* data, as a machine would typically enter a faulty condition after it has been operating normally. Lastly, the quantity of *normal* and *abnormal* samples in the testing set are fixed, unlike the sample size in the training set.

For all multivariate techniques, the 6000 features were scaled to have zero mean and unit variance. Certain multivariate outlier detection techniques perform poorly with high-dimensional data. For OCSVN, LOF, ABOD, kNN, and MCD, principal components analysis was used to decompose the input space into the 20 covariates, which cumulatively represent 96.2% of the total variance in the original data.

## 5 Results and discussion

For every experiment, 20 runs were completed; the runs are not fully independent, but we minimized the amount of overlapping data between runs. The measures of performance include precision, recall, and F1-score. All results reported include 95% confidence intervals of the mean response. To ensure statistical relevance of our results, we also performed paired-sample t-tests. In all cases, GVFOD had statistically significantly better F1 score as compared to each competing algorithm, at the 5% significance level, when presented with 2000 training data samples.

**FIGURE 4:**
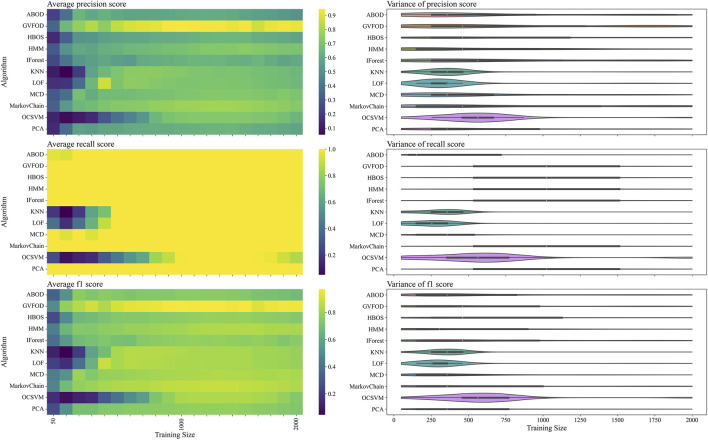
The presented figure depicts the performance of models on *tuning* data, as evaluated by three distinct metrics. The three rows of the figure illustrate the average and variance of the precision score, recall score, and f1-score, respectively, across different sizes of the training data. A heat-map is used to represent the average performance, while a violin plot is employed to visualize the variance. In the violin plot, a significant deviation from the horizontal line for each algorithm indicates a high level of variance.

The performance of GVFOD relative to other outlier detection methods on the *tuning* data is shown in Figure 4. Since this data was also used for hyperparameter tuning, the results are not indicative of true model performance. However, there are several notable peculiarities in the results.

In a machine fault detection context, precision is poor when normal operation is improperly classified as faulty, whereas recall is poor when faulty operation is improperly classified as normal.

From the recall heat-map in the second row of Figure 4, we can interpret that the vast majority of algorithms will successfully detect true machine faults. However, the precision heat-map in the first row of Figure 4 shows that it was difficult to achieve accurate classification of normal behavior. In production, a CBM system constantly reporting faults erroneously will result in excessive maintenance activity, and reduce user trust in the fault detection system (Kay et al., 2015).

Furthermore, with limited training data, model performance is degraded as expected. However, the precision heat-map in Figure 4 shows that increasing the available training data beyond 1300 periods results in reduced precision for multivariate techniques. There are two plausible explanations for this. Firstly, the hyperparameters were optimized for F1-score at a training data size of 1000. The hyperparameters could be overfit for this training size. Secondly, the *normal* training data could be non-stationary. Non-stationarity is defined as a shift in the sampling distribution of the robot arm data—as more training data accumulates, the subsequent *normal* testing data is sampled from a distribution more distant than the distribution of the initial training data.

**FIGURE 5:**
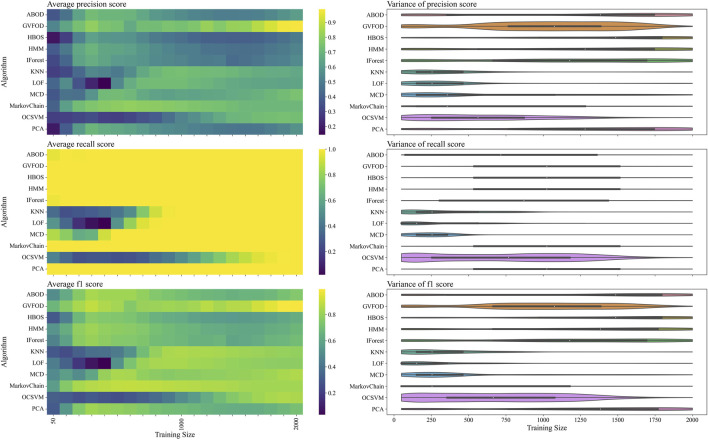
The presented figure depicts the performance of models on *evaluation* data, as evaluated by three distinct metrics. For more information about each figure, please refer to the caption of Figure 4.

To determine which of the two proposed factors more likely caused the observed drop in performance at larger training sizes, the model is evaluated on the *evaluation* half of the dataset in Figure 5. Again, we see that most algorithms exhibit similar recall (heat-map on the second row of Figure 5) and are better differentiated by their precision (heat-map on the first row of Figure 5). For multivariate outlier techniques, there is a reduction in precision as training data increases from 500 periods to 1500 periods. By visual inspection, it is clear that the performance of all algorithms changes significantly between Figures 4, 5. The first suspicion when observing this discrepancy is hyperparameter overfitting. However, because of unusual variation in performance as training data quantity changes (within both *tuning* and *evaluation* datasets), we suspect that there is significant non-stationary behavior in the *normal* operation of the robot arm.

To further investigate this claim, we completed experiments using default hyperparameters for the multivariate outlier detection algorithms. These default values are provided by PyOD (Zhao et al., 2019), and represent a real-world deployment of machine fault detection where no fault data is available. For GVFOD, no default parameters are provided in this paper. However, we propose some guidelines to select these parameters based upon knowledge of the machine and of reinforcement learning. To the best of our ability, we selected the hyperparameters based on these guidelines, with no manual tuning based on model performance.

• divs_per_dim: the number of tiles (or bins) to have for each dimension (sensor). The chosen value is (10, 10, 10), which corresponds to the number of tiles for *position*, *torque*, and *tension* respectively. This determines the amount of generalization: learning on one state can at most affect value function estimates of states $\frac{1}{10}=10\%$ of the sensor range in either direction.
• n_tilings (*m*): the number of offset tilings to use. It also determines the number of ones in the feature vector. The chosen value is 10. Combined with the previous choice of divs_per_dim, there is $\frac{1}{10\times 10}=1\%$ resolution in each dimension.
• discount_rate (*γ*): The chosen value is 0.9. This yields a pseudo-horizon of the GVF predictions of $\frac{1}{1-0.9}=10$ time-steps, and the discounted sum (and the value function) will be roughly ten times the scale of the sensor value (cumulant).
• step_size (*α*): the amount of correction with each visit to a state. The chosen value is 0.001. If there is no noise, and the true value function is fully representable with the feature mapping, the ideal value is always *m* ^−1^. With a step_size of $\frac{1}{m}\times \frac{1}{100}$ , the error would decay to zero in 100 visits to that state if the initial magnitude of value estimate updates are maintained.
• lambda (*λ*): the trace-decay parameter. The chosen value was 0.1. This value is typically fairly arbitrary - larger values pass more information backwards in time to update previously visited states, but have higher variance.
• beta (*β*): the “window width” for UDE. The chosen value was 250. This should be set to a value representative of how many time steps it would take to recognize an outlier. Since the period of the robot arm is 2000 steps, a choice of 250 is reasonable.

The Markov chain and HMM models also do not have default hyperparameters. For Markov Chain, divs_per_dim, was kept the same as its optimized value of (8, 8, 8). This results in 8^3^ = 512 states. By our judgment, this is a reasonable expert-selected value for our robot-arm application. Likewise, the hyperparameter for HMM was kept the same (8 internal states), due to computational constraints. This is supported by the algorithm runtimes in Table 4. The computations were performed using a system with an AMD Ryzen 7 4800H CPU with a 2.90 GHz clock rate, 8 MB of shared L3 cache and 16 GB DDR4-3200 RAM.

**TABLE 4:** Algorithm runtime. The median time is reported, using tuned hyperparameters.

Algorithm	Training data (samples)		Testing data (samples) 700
	700	1400	
	Time (s)		
IForest	2.26	5.44	0.89
OCSVM	0.03	0.13	0.01
LOF	0.05	0.12	0.04
ABOD	38.51	76.93	38.15
KNN	0.04	0.11	0.11
HBOS	1.45	1.82	0.11
MCD	0.69	0.84	0.00
PCA	0.35	0.75	0.04
MarkovChain	5.98	11.84	4.08
HMM	980.09	1959.79	7.99
GVFOD	2.14	4.35	0.95

**FIGURE 6:**
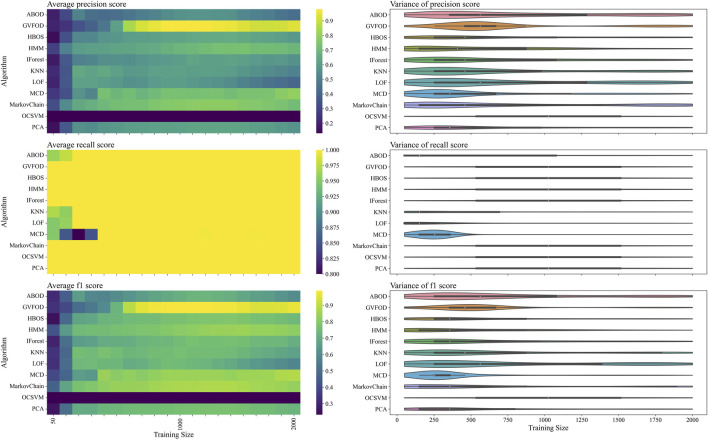
The presented figure depicts the performance of models with default hyperparameters on *tuning* data, as evaluated by three distinct metrics. For more information about each figure, please refer to the caption of Figure 4.

**FIGURE 7:**
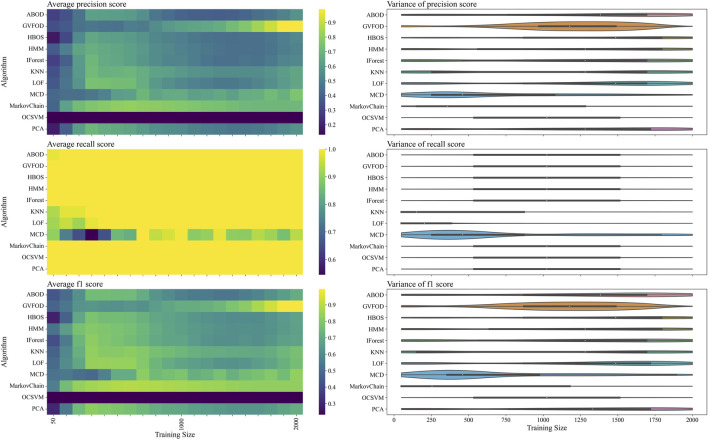
The presented figure depicts the performance of models with default hyperparameters on *evaluation* data, as evaluated by three distinct metrics. For more information about each figure, please refer to the caption of Figure 4.

The same comparisons between algorithms on the two datasets are shown in Figures 6, 7. Again, we see that there is a significant difference in performance between the two datasets. Since no parameter fitting was done, we can confidently conclude that there is a significant shift in the sampling distribution of normal data over time.

In general, multivariate outlier detection algorithms behaved as expected when comparing optimized and default parameters. LOF, kNN, OCSVM, and MCD exceed mean F1 of 80% at a training data size of 1000 periods using optimized parameters, but do not exceed 80% at any training size using default parameters.

Interestingly, the expert-selected parameters for GVFOD were better than the optimized values. Due to computational constraints, GVFOD hyperparameter tuning was only allocated 400 evaluations of the objective function. The objective is the mean F1-score with a training data size of 1000 periods, on the *tuning* dataset. The optimized parameters reached mean F1 of 96.8%, while the expert selected parameters yielded a mean F1 of 98.8%^3^. Because of the intuitive nature of GVFOD hyperparameters, they can be well-matched to a machine given machine knowledge (such as sampling frequency, number of sensors, period, etc.), outperforming optimization algorithms, even though the optimization algorithm is given access to fault data.

In the precision heat-maps of Figures 5, 7, we see that the mean precision of GVFOD increases when the availability of training data increases. Other algorithms do not always exhibit this behavior. One hypothesis to explain this behavior lies within the design of the TD(*λ*) learning algorithm. TD(*λ*) has a constant step-size parameter. Because of this, it will tend to overwrite previously learned state-value estimates as it learns, effectively assigning larger weights to data later in the training process. This *tracking* behavior allows it to better adapt to non-stationary operating data. It also results in an algorithm that better classifies normal data in the testing phase, since the *normal* testing data is collected immediately after the training data. Moreover, because the distribution of GVFOD outlier scores are calculated using the biased model, GVFOD learns not only normal behavior - but the range of non-stationarity that is acceptable in normal behavior. This conclusively explains why GVFOD achieves better precision (with no penalty to recall) compared to multivariate outlier detection methods on this dataset, when allocated large amounts of training data.

We see that GVFOD has higher variance in precision compared to most other algorithms through Figures 5, 7. This is especially apparent comparing the performance of Markov Chain outlier detection to GVFOD—both are state-space time series models, while Markov Chain is significantly more consistent, both in variation within a training data quantity, and between training data quantities. This can be a significant problem for GVFOD in practice, since high variance can manifest as inconsistent fault detection performance. However, given sufficient training data for our robot-arm (e.g. beyond 1500 training samples), we observe consistently better precision than other algorithms, which we believe to be an acceptable compromise for less consistent behavior with less training data.

## 6 Future work

GVFOD is presented here as a batch algorithm, as it requires two passes over the training data in order to learn the GVFs. A preliminary online and incremental version of GVFOD has been derived, but it is currently inapplicable to all but the smallest datasets due to computational constraints. As presented in this paper, GVFOD can only be applied used in situations where a machine starts operating in known-good conditions. An online implementation of GVFOD could be applied more broadly to situations where fault detection is required from system startup - but this would not alleviate concerns of large data requirements before reaching acceptable levels of precision and recall.

GVFOD is a general algorithm and not specifically tailored to the data in this paper. As it learns shifts in the data distribution, we expect it to generalize well to other applications and data sets, including aperiodic time series data. This intuition needs to be validated in future work by using data from other domains. Furthermore, we are applying GVFOD to simulated environments where failure can be controlled precisely, in order to evaluate how the algorithm performs in gradual-failure scenarios.

## 7 Conclusion

In this paper we used General Value Functions (GVFs) as a means to detect outliers in robotic data. The data is collected from a robot that emulates an industrial actuator—a machine that is widespread over various applications in daily life as well as in industry. We compared our new algorithm, called General Value Function Outlier Detection (GVFOD), to existing methods for outlier detection in multivariate data. As GVFOD uses reinforcement learning methods, we expect it to be better suited for time-series data than traditional methods. GVFOD exhibits higher precision paired with a comparable recall as existing methods, making it a more suited approach. While GVFOD has a high variance when only trained on a small data set, its performance significantly improves with more data. Overall, GVFOD outperforms the traditional outlier detection algorithms. As the hyperparameters are intuitive to select based on knowledge about the application, this algorithm is a viable solution to allow for condition-based maintenance in industrial applications. The results in this paper demonstrate how predictions, learned via reinforcement learning methods, can be used in real-world applications. To the best of our knowledge, this paper is the first demonstration of General Value Functions to be used for outlier detection in an industrial setting. With its improved performance when compared to traditional outlier detection methods GVFOD allows for better predictive maintenance, saving resources and cost in the process.

## Data Availability

The complete dataset and results of hyperparameter search can be found at https://figshare.com/s/4db1bbe92998aaf34323. The code to use GVFOD and to reproduce these experiments is at https://github.com/andywong36/GVFOD.

## Footnotes

1 The complete dataset and results of hyperparameter search can be found at https://figshare.com/s/4db1bbe92998aaf34323. The code to use GVFOD (including software requirements) to reproduce these experiments is on GitHub.

2 Due to its complex tree structure, the search space is described in the code on GitHub.

3 Reported on the *tuning* data, due to the confounding nature of non-stationarity on the *evaluation* data.
